# The Effect of Kyolic Aged Garlic Extract on Gut Microbiota, Inflammation, and Cardiovascular Markers in Hypertensives: The GarGIC Trial

**DOI:** 10.3389/fnut.2018.00122

**Published:** 2018-12-11

**Authors:** Karin Ried, Nikolaj Travica, Avni Sali

**Affiliations:** ^1^National Institute of Integrative Medicine, Melbourne, VIC, Australia; ^2^Discipline of General Practice, The University of Adelaide, Adelaide, SA, Australia; ^3^Faculty of Health Science and Medicine, Bond University, Gold Coast, QLD, Australia

**Keywords:** aged garlic extract, blood pressure, pulse wave velocity, arterial stiffness, inflammation, gut microbiome, B vitamins, hypertension

## Abstract

**Background:** Previous research suggests Kyolic-aged-garlic-extract to be effective in reducing blood pressure in a large proportion of hypertensive patients similar to first-line standard antihypertensive medication. High blood pressure has been linked to gut dysbiosis, with a significant decrease in microbial richness and diversity in hypertensives compared to normotensives. Furthermore, gut dysbiosis has been associated with increased inflammatory status and risk of cardiovascular events.

**Objective:** To assess the effect of Kyolic aged GARlic extract on Gut microbiota, Inflammation, and Cardiovascular markers, including blood pressure, pulse wave velocity and arterial stiffness.

**Methods:** A total of 49 participants with uncontrolled hypertension completed a double-blind randomized placebo-controlled trial of 12-weeks, investigating the effect of daily intake of aged-garlic-extract (1.2 g containing 1.2 mg S-allylcysteine) or placebo on blood pressure, pulse wave velocity and arterial stiffness, inflammatory markers, and gut microbiota.

**Results:** Mean blood pressure was significantly reduced by 10 ± 3.6 mmHg systolic and 5.4 ± 2.3 mmHg diastolic compared to placebo. Vitamin B12 status played a role in responsiveness to garlic on blood pressure in 17% of patients. Garlic significantly lowered central blood pressure, pulse pressure and arterial stiffness (*p* < 0.05). Trends observed in inflammatory markers TNF-α and IL-6 need to be confirmed in larger trials. Furthermore, aged-garlic-extract improved gut microbiota, evident by higher microbial richness and diversity with a marked increase in *Lactobacillus* and *Clostridia* species after 3 months of supplementation.

**Conclusions:** Kyolic-aged-garlic-extract is effective in reducing blood pressure in patients with uncontrolled hypertension, and has the potential to improve arterial stiffness, inflammation, and gut microbial profile. Aged-garlic-extract is highly tolerable with a high safety profile as a stand-alone or adjunctive antihypertensive treatment, with multiple benefits for cardiovascular health.

**Trial Registration:** Australian New Zealand Clinical Trial Registry ACTRN12616000185460 (https://www.anzctr.org.au/Trial/Registration/TrialReview.aspx?id=370096)

## Introduction

Our previous research suggests that Kyolic aged garlic extract is effective in reducing blood pressure in a large proportion (70–80%) of hypertensive patients similar to first-line standard antihypertensive medication (1–3), and has shown promise in reducing central blood pressure, arterial stiffness, and inflammation (3–6). A meta-analysis on the effect of garlic on blood pressure including 20 trials and more than 900 participants revealed a significant effect of garlic on blood pressure, with an average decrease in systolic blood pressure of 8.6 mm systolic and 6.1 mm diastolic in hypertensives (*n* = 14 trial arms, *n* = 468 participants) (4).

Kyolic aged garlic extract has also been shown to be effective in reducing central hemodynamic measures including central blood pressure, pulse wave velocity, pulse pressure, and arterial stiffness, which are regarded as more important predictors than peripheral blood pressure for cardiovascular disease (7, 8). Arterial stiffness, an indicator of the flexibility of the arteries, increases with age through loss of intact elastin and collagen fibers in the arterial wall, which also contributes to increased blood pressure (9). Stiffening of the arteries is a normal process of aging (10, 11), however, changes are subtle and are generally not noticeable. However, previous research has shown Kyolic aged garlic extract to be effective in reducing arterial stiffness, which in turn is related to better heart health and aerobic fitness, while the risk of cardiovascular disease is reduced (3, 8).

Previously we described the mechanism of action by which garlic influences blood pressure, involving two main signaling pathways via nitric oxide (NO) and hydrogen sulfide (H_2_S) production (12). Garlic, a sulfur donor, provides an important component for the trans-sulfuration pathway, which involves methylation and requires several co-factors such as vitamin B12, folate and vitamin B6. In addition, known genetic variants for the CBS (cystathionine-β-synthase) and CSE (cystathionine-γ-lyase) enzymes, influence the efficiency of H_2_S production, and play an important role in the susceptibility for hypertension together with deficiencies in co-factors, in particular vitamin B6. We previously identified a potentially large proportion (80%) of healthy adults in Australia with sub-optimal vitamin B12 levels (< 500 pg/l) (13). Therefore, deficiencies in co-factors, such as vitamin B12, which may serve as a proxy marker for underlying deficiencies in other B-vitamins, could be important contributors to hypertension in these individuals, which may also explain individual responsiveness to garlic supplementation seen in clinical trials.

Furthermore, high blood pressure has been linked to gut microbiota dysbiosis, both in animal and human studies with a significant decrease in microbial richness (Chao richness/relative abundance) and diversity (Shannon diversity = proportion of species/total number of species) and a significant increase in the *Firmicutes*-to-*Bacteroidetes* ratio (F/B ratio) in hypertensives compared to normotensives (14). In this small study including *n* = 10 normotensives and *n* = 7 hypertensive individuals, hypertensives had a significantly lower microbial richness and Shannon diversity (*p* < 0.05) compared to normotensives. In general, higher relative abundance/bacterial mass/microbial richness and diversity of microbial species is associated with better health (14–16).

In addition, high Firmicutes to Bacteroidetes (F/B) ratios have been associated with obesity in mice and humans (17). Also, The Firmicutes/Bacteroidetes (F/B) ratio was significantly higher in hypertensive rats compared to control rats [F/B ratio = 22.51 vs. 4.29; *p* < 0.001] (14). Natural changes in F/B ratios have been described with increasing age, the mean F/B ratio in 25–45 year old adults was higher than in the elderly of 70–90 years (*n* = 21 vs. 20: F/B ratio 12.5 vs. 2.5) (18).

Moreover, gut dysbiosis has been associated with increased inflammatory status, chronic inflammatory diseases and increased risk of cardiovascular events (19). Biochemical markers for inflammation are cytokines, which usually appear in a “cascading” system, and can take on pro- and or anti-inflammatory properties, with the balance between these cytokines, or cytokine ratios, determining the inflammatory response (20). The ratios between pro- and anti-inflammatory cytokines, such as IL-6 and IL-10 have been linked to long term outcome of acute coronary syndrome (21). Garlic has been found to increase IL-10 and inhibit TNF-α and IL-6 production in placental cell culture (22), and TNF-α in inflammatory bowel disease (23). Similarly, Kyolic aged garlic extract was found to significantly reduce the inflammatory marker TNF-alpha in hypertensive patients (3). High ratios of IL-6/ TNF-α were found relevant in the modulation of both innate and adaptive immune responses in newborns (24), and play a role in the etiology of a number of autoimmune diseases (25).

With garlic's prebiotic properties, and source of intracellular hydrogen sulfide (H_2_S) (12, 26), garlic has the potential to modulate the gut microbiota (27), and to protect from intestinal inflammation and to restore microbiota biofilm and mucus production (28). The timeframe of gut microbiota composition to change with dietary supplementation is relatively short, as shown in a 4 week study of IBS patients taking daily probiotics (29). In addition, probiotics consumption significantly reduced blood pressure, in particular in trials of longer than 8 weeks duration (meta-analysis of 9 RCTs involving 534 patients) (30).

Our 3-month randomized controlled double-blind trial (RCT) aimed to assess the effect of Kyolic aged GARlic extract on Gut microbiota, Inflammation, and Cardiovascular markers, including blood pressure, pulse wave velocity and arterial stiffness.

## Materials and Methods

### Trial Design and Participants

Adults with uncontrolled hypertension [systolic BP (SBP) ≥ 140 mmHg and/or diastolic BP (DBP) ≥ 90 mmHg] were sought to participate in the double-blind randomized placebo-controlled parallel 12-week trial investigating the effect of aged garlic extract on blood pressure, central haemodynamic measures including pulse wave velocity, inflammatory markers, and the gut microbiome. Participants were eligible, if they were on an established plan of blood pressure medication for at least 2 months, or were not taking any blood pressure medication, and their doctor was not planning to change current treatment during the trial. We excluded adults with unstable medical conditions or serious illness, at the discretion of the GP, pregnancy, severe hypertension (mean sitting SBP ≥ 180 mm Hg and DBP ≥ 110 mm Hg), those planning surgery in the next 3–4 months, or with a history of intestinal surgery, inflammatory bowel disease, celiac disease, lactose intolerance, chronic pancreatitis, or diagnosed malabsorption disorder. Additionally, participants were not enrolled, if they had been on antibiotic treatment within the last 2 months of study enrolment, taking prescribed anti-inflammatory agents, glucocorticoids, or other immune regulating prescription medication. Potential participants were advised to cease any probiotics or garlic supplementation at least 2 months prior to trial enrolment.

We recruited through the NIIM clinic newsletter, NIIM website, and flyers distributed in the local area in Melbourne. The study was approved by the NHMRC-endorsed NIIM Human Research Ethics Committee, and participants provided written informed consent.

### Allocation and Trial Supplements

Consenting eligible patients were randomly allocated to the garlic or placebo group using a computer-generated permuted random number table provided by an independent researcher not involved in patient recruitment, patient care, data collection, and follow-up.

Patients were assigned either two capsules daily of Kyolic aged garlic extract (Reserve formula; Wakunaga of America Co Ltd (31) containing 1.2 g of aged garlic extract powder and 1.2 mg S-allylcysteine (32), or to two placebo capsules containing inert microcrystalline cellulose daily for 12 weeks.

Kyolic aged garlic extract powder is manufactured from organically grown garlic bulbs, which have undergone a 20-month natural aging process at room temperature. During the aging process volatile sulfur-components found in raw garlic, such as allicin, are chemically converted into stable and standardisable components, including the main vaso-active component S-allylcysteine (SAC), associated with its blood pressure reducing properties (31–33). Garlic bulbs and Kyolic garlic extract powder, also contain insoluble dietary fiber, also known as prebiotics, acting as “food” for the good bacteria in the gut.

Placebo capsules were matched in appearance to the active capsules, and packaged in identical containers by the manufacturers off-site. Activated carbon-sachets were added to each container to disguise any odor. Patients, investigators and research assistants were blinded to treatment allocation. Blinding success of patients was assessed at the end of the trial by questionnaire.

Patients' appointments were made at NIIM 4-weekly for blood pressure and cardiovascular measurements. Eligible participants were required to fast (6–8 h) for blood testing at baseline and 12 weeks. A collection kit was provided for stool microbial analysis at baseline and 12 weeks.

Participants were instructed to start the trial capsules after blood and stool collection at baseline, and to continue until the day of stool collection at 12 weeks. Patients were given a 4-week supply of trial capsules at the baseline, 4 and 8 weeks appointments.

Participants were advised to take the trial capsules in the evening with food to minimize belching. Participants were reminded to take their usual prescription medication as instructed by their doctor, and not to alter their general diet and exercise regimen throughout the trial. Compliance was assessed by questionnaire and by pill count at the end of each visit.

### Assessments

#### Baseline Demographics and Tolerability

The baseline questionnaire assessed patient's demographics, BP and other medications, supplements, family history of cardiovascular and chronic diseases. Tolerability of trial capsules was assessed at each 4-weekly appointment using our previously developed questionnaire (1, 2). The 12-week questionnaire audited medication, and assessed acceptability of trial capsules and blinding success.

#### Clinical Blood Pressure Monitoring

Primary outcome measures were SBP and DBP at 4, 8, and 12 weeks compared with baseline. BP was measured by a trained research assistant using two independent devices, (a) a calibrated and validated digital sphygmomanometer (Omron HEM-907, JA-Davey-Pty-Ltd, Melbourne, Australia), and, (b) an oscillometric ambulatory BP monitor (Mobil-O-Graph, IEM-GmbH, Germany), with appropriate sized brachial cuffs.

The displays of the sphygmomanometers was positioned away from the patient to assure blinding to the BP readings. BP measurement was taken with the patient in seated position with the arm supported at heart level, after 5 min rest, after abstinence from food, nutritional supplements, caffeinated beverages, and smoking for a minimum of 2 h prior to BP measurement at Approximately the same time/day of the week, as per standard BP measurement guidelines (34). BP was recorded as three serial measurements at intervals of 30 s. The mean of the three BP measurements was used in the analysis. If a BP reading deviated by more than 10 mm Hg from the average reading, the BP reading on that arm was repeated.

#### Central Hemodynamic Measures, Arterial Stiffness

With the Mobil-O-Graph device we also assessed central hemodynamic measures, including central blood pressure, pulse-wave-velocity (PWV), pulse pressure, and arterial stiffness at baseline, 4, 8, and 12 weeks. The Mobil-O-Graph, used in our previous trial (3), is a brachial non-invasive device which takes a 10 s snapshot of the radial arterial pressure wave and derives the ascending aortic pressure wave, providing central cardiovascular measurements. Here PWV is indirectly derived by a computerized algorithm calculated from the brachial pulse. PWV data is provided in m/s and age-and-gender-adjusted-PWV is compared to the reference population in percent (%) (10, 11).

As PWV, a measure for arterial stiffness and a strong predictor of cardiovascular morbidity, is more accurately measured directly by the assessment of the distance traveled over time from carotid to femoral artery 7, we also used the validated SphygmoCor Xcel (device Atcor Medical, AUS) for PWV. Here, the patient is in a supine position, and the distance from the carotid to femoral pulse is measured, and the time for the pulse wave to travel from carotid to femoral artery is assessed by applanation tonometry.

#### Blood Tests

The research assistant, trained in phlebotomy, took a fasted blood sample at baseline and 12 weeks to assess the red blood cell active Vitamin B12 and inflammatory markers TNF-α and interleukin-6 (IL-6).

#### Stool Test/Gut Microbiota

Enrolled patients were provided with a commercially available test kit from Genova Diagnostics, USA (www.gtx.net) at baseline and at 12 weeks, and were instructed to collect a stool sample within a few days of their baseline appointment and to send this to Genova Diagnostics for a Microbial Ecological Profile (GI-Effects-Stool-Profile), providing a validated comprehensive profile of commensal bacterial species in colony-forming-units (CFU)/g stool by multiplex PCR-DNA analysis. The report was used to calculate Relative Abundance (RA)/ microbial richness, Shannon diversity, and Firmicutes/Bacteroidetes ratio.

### Statistical Analysis

Primary outcome measures were blood pressure change over time (SBP/DBP) at 12 weeks compared to baseline, and secondary outcome measures were central hemodynamic measures, including pulse wave velocity, changes in gut microbiota measured by relative abundance/ microbial richness, microbial diversity, and Firmicute/Bacteroidetes ratio, and changes in inflammatory markers TNF-α and IL-6.

Vitamin B12 blood levels were explored as potential confounding variables.

Analyses were performed using IBM SPSS version 24. Statistical significance was set at *p* < 0.05.

Descriptive analysis was carried out for baseline variables. Normality of data distribution was assessed by the Shapiro–Wilks test. Differences between the groups at 12 weeks compared to baseline were assessed by chi-square test for binominal variables, and by one-way ANOVA with Bonferroni adjustment for continuous and normally distributed variables. Potential confounding variables were included in the analysis using ANCOVA (analysis of co-variance), e.g., baseline blood pressure, or age and gender were relevant for central hemodynamic measures, e.g., pulse wave velocity. Primary analysis was conducted with all participants following protocol, excluding data points owing to BP medication change. Subgroup analyses of changes in SBP/DBP overtime by BP medication at baseline were also undertaken.

We compared Vitamin B12 levels to BP change between groups by scatterplots. As previously described, we defined responders to garlic as mean BP reduction by more than 3% in SBP (≥5 mm Hg) or DBP (≥3 mm Hg) over time compared to baseline, which is clinically and statistically meaningful and similar to the definitions by others (3, 35).

Changes in cytokines TNF-α and IL-6 levels were analyzed by ANOVA excluding high values/outliers due to infection (e.g., common cold), and by subgroups (≥50 and < 50% percentile). Cytokine ratios IL-6/ TNF- α were also calculated.

#### Microbial Analysis

Microbial Ecological Stool Profile was done by validated multiplex PCR by Genova Diagnostics, USA (www.gtx.net). Results were presented as colony-forming-units (CFU) per gram of stool of 21 bacterial species, grouped into four main phyla: Bacteroidetes, Firmicutes, Actinobacteria, and Proteobacteria phyla.

We calculated Relative Abundance (RA), microbial richness, or “total” bacterial mass as the sum of all analyzed bacteria groups in colony forming units per gram (CFU/g), calculated changes over time (12 weeks vs. baseline) by bacterial group, and compared statistical significant differences between groups by ANOVA.

Diversity of species, Shannon diversity was calculated as the sum of the number of individual species relative to the total number of bacteria using the formula: **H** **=** SUM[(pi)^*^ln(pi)] with SUM = Summation, and pi = number CFU/g in individual bacterial group/sum of all bac in CFU/g. Changes over time between the groups were assessed by ANOVA.

Correlations between BP change and gut microbiota change were analyzed by the non-parametric Spearman correlation.

#### Sample Size

A sample size of 50 patients was calculated based on the following assumptions: To detect a difference of 8 mmHg BP (SD = 9) in BP change between the active treatment (*n* = 25) and control (*n* = 25) with 80% power and 95% confidence (3, 4); to account for 10% drop-out or non-attendance at all appointments.

## Results

### Participants

The trial was conducted in Melbourne, Australia between May 2016 and October 2017. Patients were recruited through the NIIM clinic's doctors, the NIIM newsletter and website, and letterbox drop of flyers.

**Figure 1:**
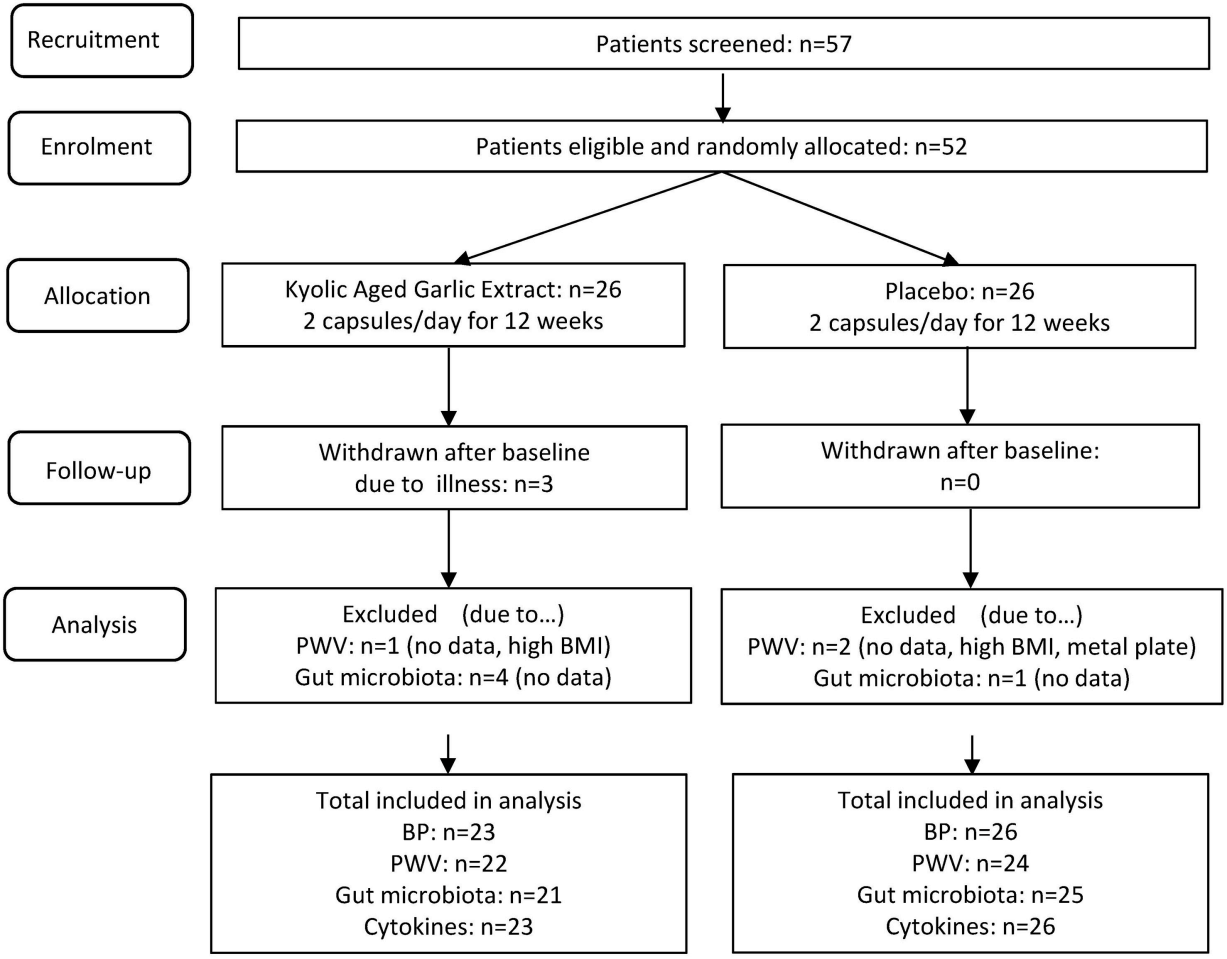
GarGIC trial flow chart.

A total of 57 patients were screened for eligibility, 52 patients were enrolled in the trial and randomly allocated to the garlic or the placebo group. Three patients withdrew due to illness unrelated to the trial, with a total of 49 patients completing the trial in Dec 2016. Full data analysis was available for all outcome measures by October 2017, with some missing data due to a variety of reasons outlined in Figure 1.

Baseline characteristics included age, gender, BMI, smoking habits, family history of cardiovascular diseases, Vitamin B12, SBP and DBP, and blood pressure medication. All but baseline SBP (*p* = 0.05) were not significantly different between the groups (Table 1). Potential confounding factors, including baseline SBP, were accounted for in the analyses.

**Table 1:** Baseline characteristics.

**Demographics**	**All (*n* = 49)**	**Garlic (*n* = 23)**	**Placebo (*n* = 26)**	***p*-value**
	**Mean (SD)**	**Mean (SD)**	**Mean (SD)**	
Age (years)	62.3 (10.5)	62.8 (9.3)	61.9 (11.8)	ns
BMI (kg/m^2^)	29 (7)	28.6 (7.7)	29.7 (6.2)	ns
Vitamin B12 (pg/ml)	517.9 (273)	534 (323.4)	503.6 (225.4)	ns
SBP (mmHg)	148.6 (15.8)	153.3 (16.4)	144.3 (14.2)	0.05
DBP (mmHg)	91.4 (12)	93 (10.9)	90 (12.3)	ns
	***N*** **(%)**	***N*** **(%)**	***N*** **(%)**	
Male/female	22/27 (45/55)	10/13 (44/56)	12/14 (46/54)	ns
Current smoker	3 (6)	3 (13)	0 (0)	ns
Family history of CVD	30 (61)	13 (57)	17 (65)	ns
Heart attack	9 (18)	4 (18)	5 (19)	ns
Stroke	9 (18)	5 (21)	4 (15)	ns
CAD, bypass	1 (2)	1 (4)	0 (0)	ns
Hypertension	16 (33)	6 (26)	10 (38)	ns
**BP MEDICATION**				
Yes	31 (63)	14 (61)	17 (65)	ns
BP Medication, *n*				
0	18 (37)	9 (39)	9 (35)	ns
1	29 (59)	14 (61)	15 (58)	ns
2	1 (2)	0 (0)	1 (4)	ns
3	1 (2)	0 (0)	1 (4)	ns
**BP-MEDICATION TYPE**				
ACEI	23 (47)	13 (57)	10 (39)	ns
A2RA	7 (14)	2 (9)	5 (19)	
CCB	1 (2)	0 (0)	1 (3.8)	
BB	1 (2)	0 (0)	1 (3.8)	
D	1 (2)	1 (4.3)	0 (0)	
**OTHER MEDICATIONS**				
Yes	23 (47)	13 (57)	10 (39)	
Blood- thinning	9 (18)	7 (30)	2 (8)	
Lipid (statin)	8 (16)	5 (22)	3 (12)	
Diabetes	3 (6)	2 (8)	1 (4)	
Depression/mental health	2 (4)	1 (4)	1 (4)	
Reflux/PPI	5 (10)	3 (13)	2 (8)	
Other Medications	10 (20)	2 (9)	8 (31)	

*BMI, body mass index; SBP, systolic blood pressure; DBP diastolic blood pressure; CVD, cardiovascular disease; CAD, coronary artery disease; ACEI, angiotensin converting enzyme inhibitor; A2RA, angiotensin II receptor blocker; CCB, calcium channel blockers; BB, beta blockers; D, diuretics; PPI, proton pump inhibitors; N, number; SD, standard deviation*.

Participants, males/females (45/55%), were of 62 ± 10 years of age with a high average BMI of 29 ± 7 kg/m^2^. Family history of cardiovascular events was reported by 60% of the participants.

Two-thirds of participants took standard blood pressure medication (63%), most took only one type (59%), with angiotensin-converting-enzyme-inhibitors (ACEI) being the most often prescribed type (47%). Of those participants all but one (*n* = 1) did not change their blood pressure regimen throughout the trial. About half of the participants took other prescription medications, and did not change these over the period of the trial (Table 1). None of the participants reported a substantial change in diet or exercise regimen throughout the trial period.

### Blood Pressure and Central Hemodynamic Measures

Our sample was a homogenous group of people with uncontrolled hypertension and of similar age. Testing for normality of data distribution by Shapiro–Wilk test confirmed normal distribution of SBP, DBP and hemodynamic markers (PWV, MAP, PP, cSBP, cDBP, cPP), in the garlic and the placebo groups.

Analysis of all participants (*n* = 49) revealed a reduction in SBP over 12 weeks within the garlic group (−14.3 ± 13.9 mmHg) compared to placebo (−4.3 ± 11.4 mmHg), resulting in a mean difference SBP_change_ (SE) of −10.0 ± 3.6 mmHg between the groups with high statistical significance (*p* = 0.008).

**Figure 2:**
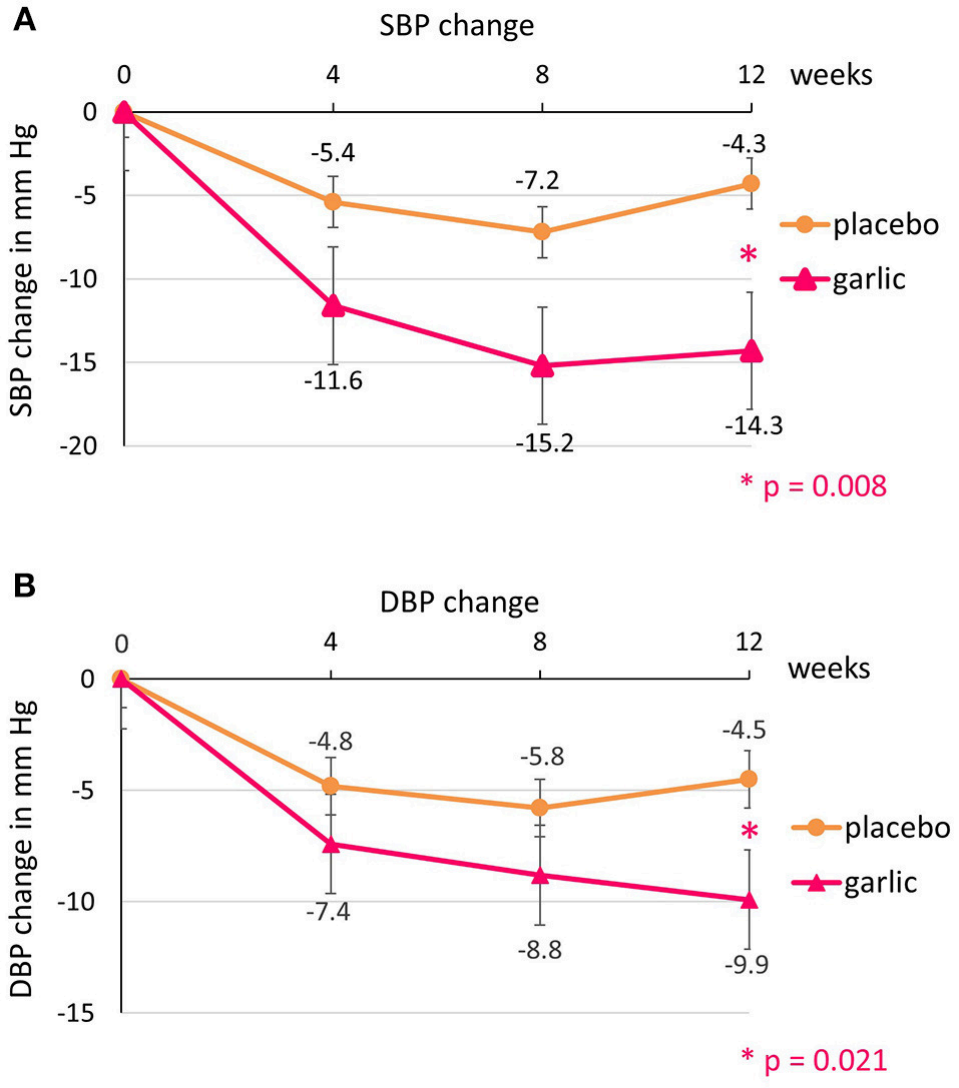
Blood pressure changes over time by group: **(A)** systolic blood pressure (SBP) and **(B)** diastolic blood pressure (DBP).

Diastolic blood pressure (DBP) was reduced on average by 9.9 ± 6.8 mmHg within the garlic group compared to 4.4 ± 8.9 mmHg in the placebo group, providing a mean difference DBP_change_ (SE) of −5.4 ± 2.3 mmHg with statistical significance (*p* = 0.02) (Figure 2).

The trajectory of blood pressure change over time within both groups is in line with the findings in previous trials (1–3), whereby blood pressure dropped slightly from baseline in the placebo group due to reduced whitecoat effect and familiarization with the trial staff and environment (36–38), but did not decrease to the same extent as in the garlic group, resulting in a statistically significant mean BP difference between the groups.

Subgroup analysis of participants taking standard BP medication (*n* = 16/17) revealed a slightly greater reduction in SBP of 12.3 ± 4.7 mmHg compared with participants not on BP medication (*n* = 7/9) of 4.9 ± 5.4 mmHg (*p* = 0.014) (Table 2). However, the small number in the subgroup of participants not taking standard BP medication limited interpretability of findings.

**Table 2:** Blood pressure and central hemodynamic measures.

								**Garlic vs. Placebo**	
					**Baseline**	**12 weeks**	**Within group**	**Between groups**	
	**Variable**	**Unit**	**Group**	***N***	**Mean (SD)**	**Mean (SD)**	**Mean change (SD)**	**Mean diff (SE)**	***P*****-value**
All	SBP	mm Hg	Garlic	23	153.3 (16.4)	139 (15.1)	−14.3 (13.9)	−10 (3.6)	0.008
			Placebo	26	144.3 (14.2)	140 (16.3)	−4.3 (11.4)		
Subgroups	BPmed		Garlic	16	156 (12)	139.5 (12.2)	−16.5 (15.4)	−12.3 (4.7)	0.014
			Placebo	17	142.8 (13.7)	138.6 (15.2)	−4.2 (11.5)		
	No BPmed		g	7	147.3 (23.8)	137.9 (21.4)	−9.4 (8.9)	−4.9 (5.4)	0.38
			p	9	147.2 (15.5)	142.7 (18.8)	−4.6 (11.8)		
All	DBP	mm Hg	Garlic	23	93 (10.9)	83.1 (10.4)	−9.9 (6.8)	−5.4 (2.3)	0.02
			p	26	90 (12.3)	85.5 (13)	−4.4 (8.9)		
Subgroups	BPmed		Garlic	16	94 (10.9)	83.4 (11.2)	−10.6 (7.6)	−4.7 (2.9)	0.1
			Placebo	17	89.9 (13.8)	84 (11.9)	−5.9 (9.2)		
	No BPmed		g	7	90.7 (11.3)	82.4 (9.2)	−8.3 (4.9)	−6.7 (3.4)	0.07
			p	9	90 (11.8)	88.4 (15.2)	−1.6 (8)		
All	PP	mm Hg	Garlic	23	60.3 (16.8)	54.8 (10.7)	−5.5 (13)	−9.6 (3.4)	0.008
			p	26	51.1 (12.7)	55 (11.3)	4.1 (9.9)		
	cSBP	mm Hg	Garlic	22	140.8 (17.3)	126.8 (13.5)	−14 (14)	−12.5 (4.6)	0.01
			p	24	129.7 (15.1)	128.3 (16.2)	−1.4 (17.2)		
	cDBP	mm Hg	Garlic	22	95.3 (12.3)	84.7 (10.2)	−10.6 (8.7)	−6.1 (3.2)	0.06
			p	24	92.6 (14.9)	88.2 (15)	−4.5 (12.5)		
	cPP	mm Hg	Garlic	22	45.4 (13.6)	42.3 (9)	−3.1 (10.7)	−6.5 (3.0)	0.04
			p	24	37.2 (9.3)	40.5 (7.3)	3.4 (9.6)		
	AP	mm Hg	Garlic	22	15.1 (9.4)	13.4 (7.9)	−1.7 (7.1)	−3.1 (2)	0.13
			p	24	11 (6)	12.4 (6.1)	1.4 (6.3)		
	MAP	mm Hg	Garlic	22	121 (13)	108.3 (11.5)	−12.6 (10.1)	−7.9 (4)	0.05
			p	24	114.3 (14)	109.6 (16.7)	−4.7 (16.4)		
	PWV_sphyg_	m/s	Garlic	23	12.8 (2.1)	12.1 (1.9)	−0.7 (1.5)	−0.7 (0.5)	0.18
			p	26	12.3 (3.3)	12.3 (3.2)	0.02 (2.1)		
	PWV_mobile_	m/s	Garlic	22	9.8 (1.6)	9.7 (1.6)	−0.1 (1.1)	−0.1 (0.3)	0.7
			p	24	9.4 (1.9)	9.2 (1.7)	−0.2 (1.1)		
Age, gender adjusted	PWV_mobilo_adj_	%	Garlic	22	72 (12) %	64 (13)%	– 8.7 (14)%	−7.7 (4)%	0.046
			p	24	63 (15)%	62 (12)%	−1 (12)%		

*N, number; Mean change, mean change over time (within group), mean at 12 weeks minus mean at 0 wks; SD, standard deviation; Mean Diff, mean difference (between groups) by AN(C)OVA; SE, standard error; BPmed, standard blood pressure medication; SBP, systolic blood pressure; DBP, diastolic blood pressure; PP, pulse pressure; cSBP, central systolic blood pressure; cDBP, central diastolic blood pressure; cPP, central pulse pressure; AP, augmentation pressure; MAP, mean arterial pressure; PWV_sphyg_, pulse wave velocity measured with the SphygmoCor device; PWV_mobilo_, pulse wave velocity measured with the Mobil-O-Graph; PWV_mobilo_adj_, age and gender adjusted PWV provided in % compared to reference population values*.

A number of central hemodynamic measures changed significantly over time between groups, including cSBP (central systolic blood pressure; MeanDiff (SE): −12.5 (4.6) mmHg, *p* = 0.01), PP (pulse pressure; MeanDiff (SE): −9.6 (3.4) mmHg, *p* = 0.008), cPP (central pulse pressure; MeanDiff (SE): −6.5 (3.0) mmHg, *p* = 0.04), MAP (mean arterial pressure; MeanDiff (SE):−7.9 (4), *p* = 0.05), and age-and gender-adjusted pulse wave velocity [PWV_mobile_adj_ = MeanDiff (SE): −7.7 (4) %, *p* = 0.046], an indicator for arterial stiffness. PWV measured in m/s with both devices (PWV_mobilo_ and PWV_sphyg_) but not adjusted for age and gender tended to improve in the garlic group compared to placebo albeit not statistically significant. In summary, Kyolic aged garlic extract significantly lowered central blood pressure, pulse pressure and adjusted pulse wave velocity, an indicator for arterial stiffness (Table 2).

### Vitamin B12

Vitamin B12 is one of the important co-factors in the mechanism of action for blood pressure reduction amongst other B-complex vitamins and genetic factors (12). As there is a large proportion of adults in Australia below the optimal levels of Vitamin B12 (13), we explored whether a low Vitamin B12 level may be involved in the responsiveness to garlic in hypertensives.

**Figure 3:**
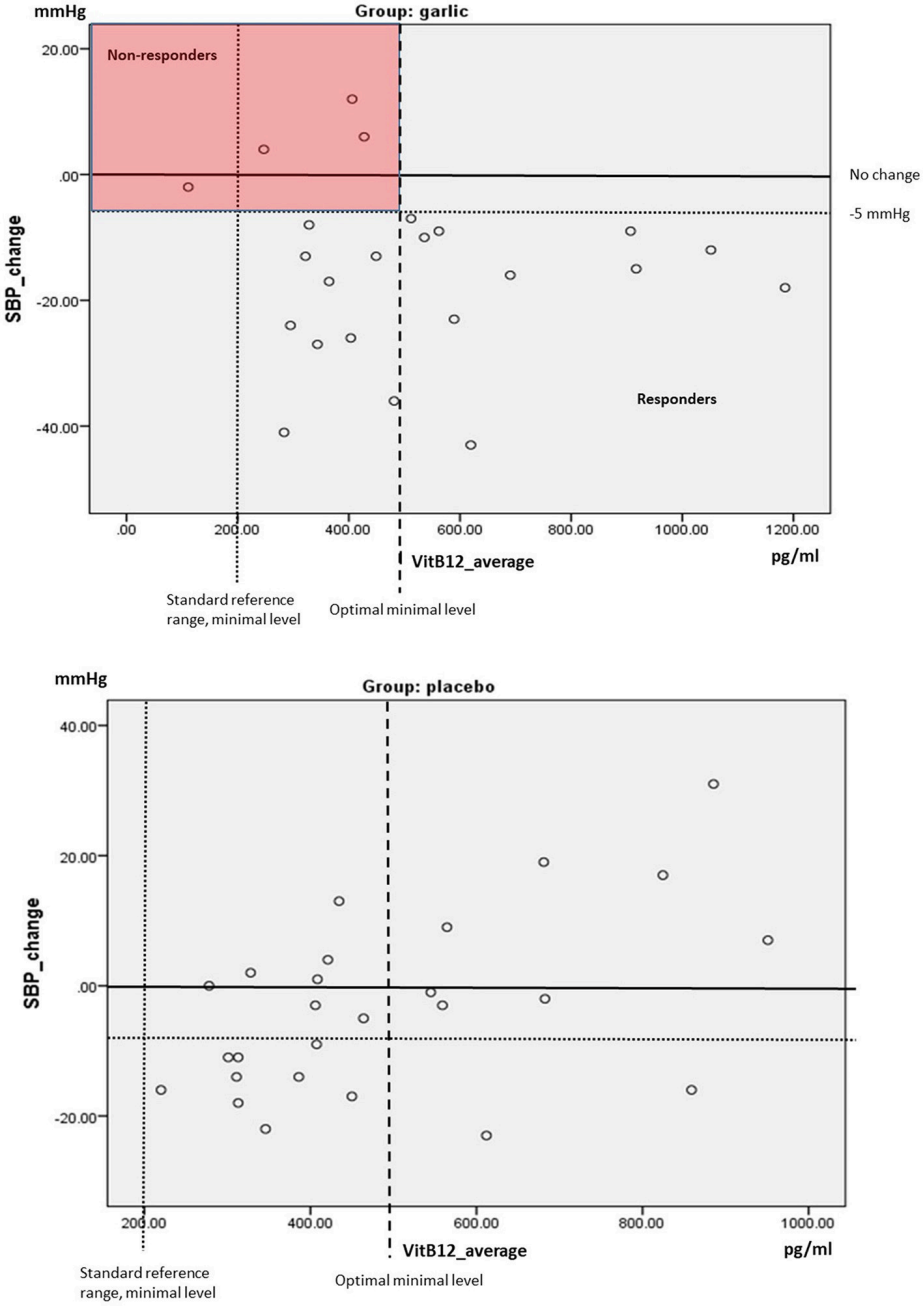
Scatterplot SBP changes over time compared to average levels of Vitamin B12 (mean levels at baseline and 12 weeks) by group. Red box highlights non-responders (SBP change < 5 mmHg).

In this study, there were 4 out of 23 (17%) non-responders in the garlic group (SBP change < 5 mmHg), and all had lower Vit B12 levels with n = 1 below the standard reference range of 200 pg/ml, and *n* = 3 had Vit B12 concentrations below the optimal level of 500 pg/ml (39) (Figure 3).

### Gut Microbiota

#### Relative Abundance/Microbial Richness

**Figure 4:**
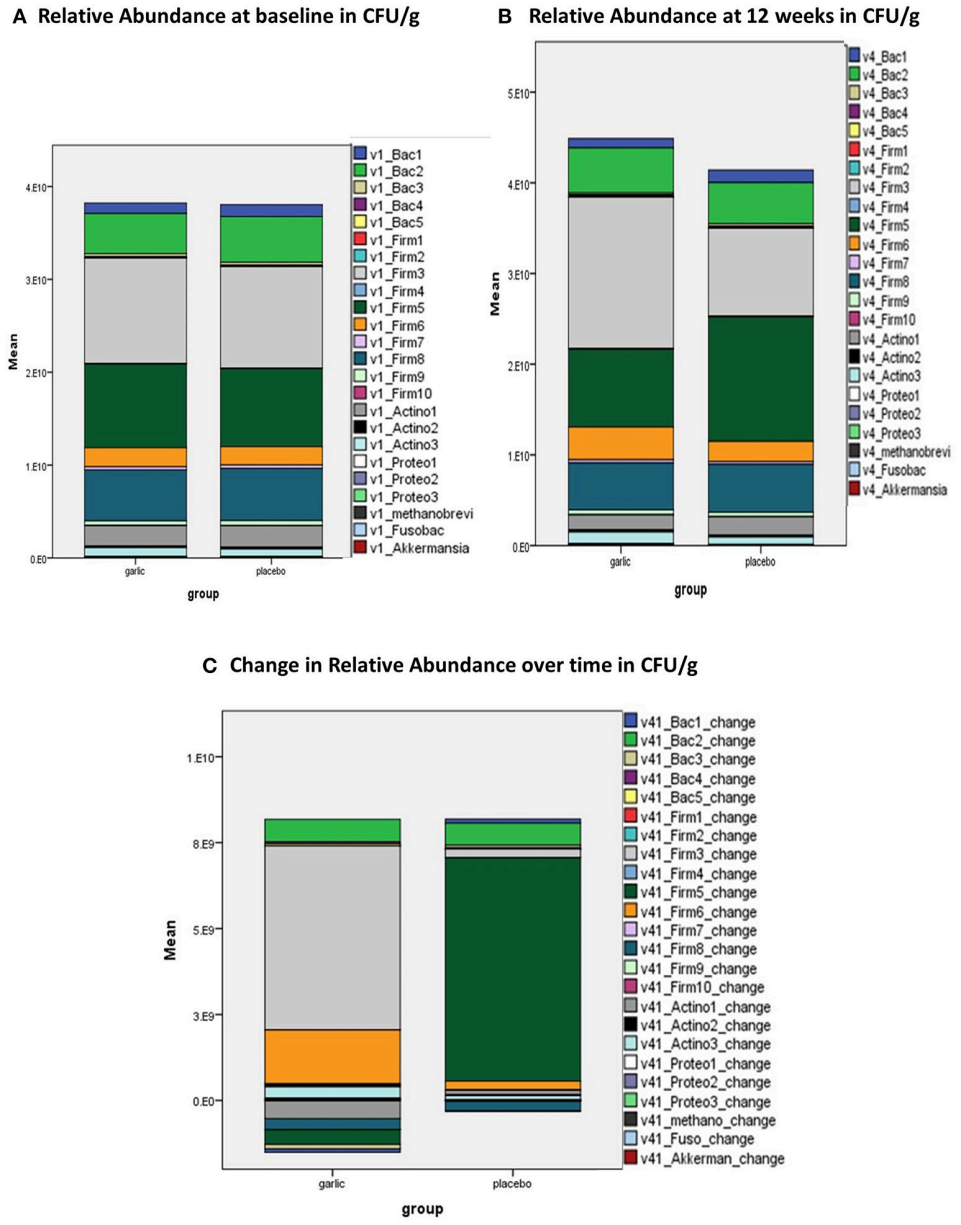
Relative Abundance in CFU/g at **(A)** baseline; **(B)** 12 weeks; and **(C)** change over time by group (garlic vs. placebo). CFU/g, colony forming units per gram stool; v1, visit 1, baseline; v4, visit 4, 12 weeks; Bac 1, Bacterioidetes-Prevotella group; Bac 2, *Bacteroides vulgatus*; Bac 3, *Barnesiella* spp.; Bac 4, *Odoribacter* spp.; Bac 5, *Prevotella* spp.; Firm 1, Anaerotruncus colihominis; Firm 2, Butyrivibrio crossotus; Firm 3, *Clostridium* spp.; Firm 4, *Coprococcus eutactus*; Firm 5, *Faecalibacterium prausnitzii*; Firm 6, *Lactobacillus* spp.; Firm 7, *Pseudoflavonifractor* spp.; Firm 8, *Roseburia* spp.; Firm 9 =; *Ruminococcus* spp.; Firm 10, *Veillonella* spp.; Actino 1, *Bifidobacterium* spp.; Actino 2, *Bifidobacterium longum*; Actino 3, Collinsella aerofaciens; Proteo 1, Desulfovibrio piger; Proteo 2, *Escherichia coli*; Proteo 3, Oxalobacter formigenes; Methanobrevi, *Methanobrevibacter smithii*; Fusobac, *Fusobacteriim* spp.; Akkermansia, *Akkermansia muciniphila*.

Relative Abundance or total bacterial mass was calculated as the sum of 21 bacteria groups including the four main phyla of Bacterioidetes, Firmicutes, Actinobacteria, and Proteobacteria in CFU/g. Figure 4 illustrates the relative abundance at baseline (Figure 4A) and at 12 weeks (Figure 4B) in both groups as stacked bar chart, and Figure 4C illustrates the change in relative abundance over time by group. Interestingly, there was a marked increase of *Lactobacillus* (Firm6) bacteria and *Clostridia* species (Firm3) in the garlic group, while *Faecalibacterium prausnitzii* (Firm5) markedly increased in the placebo group. All marked observed changes were in the Firmicutes phylum albeit not statistically significant between the groups (Figure 4, Table S1).

#### Microbial Diversity

**Figure 5:**
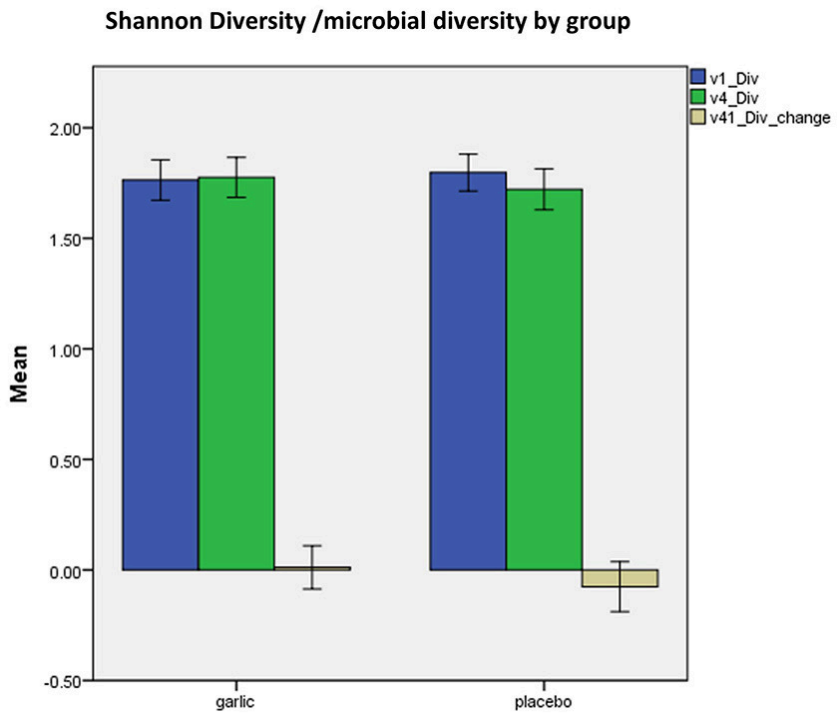
Shannon microbial diversity at baseline (blue), 12 weeks (green), and change over time (beige) by group (garlic vs. placebo). v1, baseline; v4, 12 weeks; Div, Shannon diversity, error bars 95%CI.

There was a small but statistically insignificant shift in microbial diversity with an increase in diversity in the garlic group, while there was a small decrease in diversity in the placebo group over time (Figure 5).

#### Firmicutes/Bacteroidetes Ratio

Comparison of the Firmicutes/Bacteroidetes Ratio between the groups revealed a small decrease in the garlic group and a small increase in the placebo group over time (Table 3). Subgroup analysis of those with a high (≥14) or low F/B ratio (< 14) at baseline revealed a trend in the garlic group improving more than the placebo group in the high F/B subgroup toward a lower F/B ratio (garlic: mean ± SD = −6.8 ± 19.8 vs. placebo: mean ± SD = −0.4 ± 12.1).

**Table 3:** Firmicutes/Bacteroidetes (F/B) Ratio.

**Participants**	**Variable**	**Group**	***N***	**Baseline**	**12 weeks**	**Within group**	**Between groups**
				**mean (SD)**	**mean (SD)**	**mean diff (SE)**	***p*****-value**
All	F/B ratio	garlic	21	29.2 (21.5)	27.6 (16.1)	−1.6 (21.9)	ns
		placebo	24	17 (7.1)	20.6 (12.1)	2.9 (12.2)	
Subgroup	Low F/B ratio (< 14 at BL)	garlic	5	11.2 (3.0)	28.8 (20.0)	17.6 (19.0)	ns
		placebo	6	7.2 (2.1)	17.8 (9.7)	10.7 (9.2)	
	High F/B ratio (> = 14 at BL)	garlic	16	34.8 (21.8)	27.3 (15.4)	−7.6 (19.5)	ns
		placebo	18	21.2 (5.3)	21.5 (12.9)	0.3 (12.1)	

*BL, baseline*.

### Correlations Blood Pressure–Microbiota

To assess whether there were any statistically significant correlations between changes in blood pressure and microbiota, we used Spearman correlation.

No significant correlations were obtained between BP and observed microbiotic changes within the groups. However, the observed changes in the Firmicutes phylum in the garlic group with an increase in numbers in *Clostridia* and *Lactobacillus* species and a decrease in *F. prausnitzii*, were significantly correlated (*Lactobacillus* and *Clostridia*: *r* = 0.561, *p* = 0.01); and *Clostridia* and *F. prausnitzii*: *r* = 0.639, *p* = 0.02).

Observed microbiotic changes in the placebo group followed an inverse direction compared to the garlic group, while *F. prausnitzii* increased, the *Clostridia and Lactobacillus* species decreased over time, with a significant correlation between *Clostridia* decrease and *F. prausnitzii* increase (*r* = 0.619, *p* = 0.01).

### Inflammatory Markers/Cytokines

Cytokines TNF-α and IL-6 were investigated. After excluding outliers, e.g., high values in participants presenting with an acute infection (common cold), we observed a trend toward a greater decrease in inflammatory marker TNF-α over time in the garlic group compared to the placebo group, albeit statistically not significant (Table 4). In contrast, a greater decrease but not statistically significant decrease in IL-6 was observed in the placebo group.

**Table 4:** Cytokines.

**Participants**	**Variable**	**Group**	***N***	**Baseline**	**12 weeks**	**Within group**	**Between groups**
**All**				**Mean (SD)**	**Mean (SD)**	**Mean diff (SE)**	***p*****-value**
				fg/ml	fg/ml	fg/ml	
	TNFα	Garlic	23	193.4(160.3)	116.2(139.7)	−77.2(225.8)	ns
Excl outliers *n* = 3		Placebo	23	194.2(168.9)	125(151.8)	−69.2(139.9)	
Excl outliers *n* = 4	IL6	Garlic	19	935.5(927)	768.2(877.6)	−167.3(283)	ns
Excl outliers *n* = 2		Placebo	24	1109(1269)	547.2(834)	−562(208.5)	
	TNFα/IL6	Garlic	19	11.8(34.8)	21.1(63.0)	9.2(8.4)	ns
		Placebo	23	8.9(35.9)	44.1(97.9)	35.1(19.5)	
Subgroups	TNFα, low BL < 200 fg/ml	Garlic	12	102.1(71.5)	173.8(166.5)	71.7(176.4)	ns
		Placebo	14	93.1(78.8)	71.8(57.1)	−21.3(97.0)	
	TNFα, high BL 200–900 fg/ml	Garlic	11	293.0(173.3)	53.4(64.0)	−239.6(149.1)	ns
		Placebo	9	351.5(150.6)	207.7(213.2)	−143.8(168.3)	
	IL6 low BL < 1,000 fg/ml	Garlic	13	570(344)	349(363)	-221(444)	ns
		Placebo	13	266(274)	52(140)	−213(319)	
	IL6 high BL >1,000–2,200 fg/ml	Garlic	6	1, 727(1309)	1, 676(1, 006)	−52(2, 229)	ns
		Placebo	11	2, 106(1263)	1, 132(940)	−974(1, 391)	

*TNFα, tumor necrosis factor alpha; IL6, interleukin 6; BL, baseline; excl, excluding, fg/ml, femtograms/milliliter; ns, not significant*.

As each cytokine influences levels of other cytokines, and IL-6 has been shown to inhibit TNF-α (40), we also calculated the TNF-α/IL6 ratio. A smaller ratio was observed in the garlic group (TNF-α/IL6 = 9.22 ± 8.4) compared to placebo (TNF-α/IL6 = 35.1 ± 19.5), which can be regarded as a beneficial trend (Table 4).

In subsequent subgroup analyses we compared groups with high or low levels of inflammatory markers at baseline.

For TNF-α we divided into two even subgroups (~50% percentile), low < 200 fg/ml and those above 200 fg/ml. A greater reduction in the subgroup with high TNF-α levels was observed in the garlic group compared to placebo, albeit not statistically significant [mean diff (SE) = −95.8 (71) fg/ml, *p* = 0.2] (Table 4). For IL-6 we divided participants into two even subgroups (~50% percentile), high levels ≥ 1,000 fg/ml, and low < 1,000 fg/ml. In the subgroup with high IL-6 levels, there was a greater reduction of IL-6 in the placebo group, albeit not statistically significant (Table 4).

### Tolerability, Acceptability, and Blinding

All participants tolerated the trial capsules well, and found it very easy/easy and very acceptable/acceptable to take the capsules (garlic: 87.5/12.5%; placebo: 92/8%). No participants experienced any adverse effects, and only few mentioned garlic taste and burping in the first week of the trial (*n* = 5), but no participant found this bothersome.

Blinding was successful, with two-thirds (66.5%) in the garlic group and almost 85% in the placebo group being unsure or incorrect in guessing their allocated group (Table 5).

**Table 5:** Blinding.

	**Garlic (*n* = 24)**		**Placebo (*n* = 26)**		***p*-value**
	***N***	**%**	***N***	**%**	
Correct	8	33.3	4	15.4	ns
Unsure	11	45.8	16	61.5	ns
Incorrect	5	20.7	6	23.1	ns

*ns, not significant*.

## Discussion

Our trial suggests Kyolic aged garlic extract to be effective in reducing blood pressure in patients with uncontrolled hypertension, in line with our previous trials (1–3). A dosage of two capsules daily containing 1.2 g of aged garlic extract containing 1.2 mg of the vaso-active S-allylcysteine (SAC) significantly lowered SBP by 10 mmHg compared to placebo over 12 weeks (*p* = 0.008), DBP by 5.4 mmHg (*p* = 0.02) in a group of 50–70 year olds, and was highly tolerated. Subgroup analysis of participants taking standard BP medication revealed a slightly greater reduction in SBP by 12.3 mmHg (*n* = 33, 68% of participants), while the low sample size in the subgroup of participants not taking standard BP medications limited interpretability.

The average blood pressure reduction by Kyolic aged garlic extract alone or in combination with other blood pressure medications, is comparable to conventional standard blood pressure drug therapies, reducing the risk of cardiovascular disease such heart attack or stroke by 30–40% (41).

In this trial, 83% of the participants responded to garlic with a SBP reduction of more than 5 mmHg, a slightly larger proportion than previously found (70%) (3).

To explore potential underlying factors of individual responsiveness to garlic supplementation, we tested for vitamin B12 levels in this trial. Vitamin B12 is one of the important co-factors implicated in the methylation and H_2_S signaling pathway by which garlic reduces blood pressure, and may serve as a proxy marker for other underlying deficiencies in vitamin B6, vitamin B2, and folate, also essential co-factors in H_2_S production (12, 42). Vitamin B12 deficiency can lead to elevated homocysteine, a marker for endothelial dysfunction and a risk factor for cardiovascular disease, due to impaired re-methylation. Elevated homocysteine may subsequently be shunted into the trans-sulfuration pathway at an increased rate, which in turn requires vitamin B6 for CBS- and CSE-enzyme dependent H_2_S production (12, 42).

While all non-responders (17%) in this trial were below the optimal level of Vit B12 (< 500 pg/ml), none of the responders were below the standard level (< 200 pg/ml), but a proportion of BP responders (40%) were between standard and optimal levels (200–500 pg/ml) of VitB12, indicating a non-exclusive involvement of vitamin B12 in the BP reducing mechanism. While vitamin B12 may play a role in the responsiveness to garlic on blood pressure, other factors, such as vitamin B6 and folate, as well as genetic factors, such as the trans-sulfuration gene cystathione-β-synthase (CBS) are also important in the biochemical mechanism (12), but were not assessed in this trial. Here, we speculate that the non-responders may have been deficient in multiple B-vitamins, including vitamin B12, while responders including those with lower non-optimal vitamin B12 levels may have had sufficient vitamin B6 levels needed for enzymatic conversion of garlic's sulfur compounds to H_2_S.

In this trial, a number of central hemodynamic measures, including central systolic blood pressure and pulse wave velocity, a primary indicator for arterial stiffness, were significantly lowered by aged garlic extract.

Central hemodynamic measures are regarded as more important predictors than peripheral blood pressure for cardiovascular disease (7). Our findings are in line with previous trials showing a beneficial effect of aged garlic extract on pulse wave velocity and endothelial function (3, 8), and provide new evidence that aged garlic extract has the potential to reduce central blood pressure and arterial stiffness in individuals with uncontrolled hypertension. As arterial stiffness increases with age by an average of 1.43 m/s PWV in 10 years (10, 11), our findings suggest Kyolic to have the potential to reverse the aging of arteries and therefore arterial stiffness by about 5 years evident by a mean reduction of PWV by 0.7 m/s within 3 months.

Additionally, elevated levels of inflammatory markers TNF-α and TNF-α/IL-6 ratio tended to decrease more in the garlic group, in line with previous trials (3, 6). Trends observed in inflammatory markers TNF-α and IL-6 need to be confirmed in larger trials.

Furthermore, comprehensive microbial stool analysis revealed an increase in microbial richness, microbial diversity, and a trend toward a healthier Firmicutes-to-Bacteroidetes ratio in the garlic group compared to placebo. Specifically, we observed a marked shift of bacterial species in the Firmicutes phylum in the garlic group compared to placebo. While aged garlic extract influenced the increase in Lactobacillus and Clostridia species, the observed shift in the placebo group was evident by an increase in *F. prausnitzii* to the detriment of *Lactobacillus* and *Clostridia* species.

*Lactobacillus* bacteria, increased by the prebiotic aged garlic extract, are generally regarded as beneficial (43), while common *Clostridia* species colonization in the gut have been found to activate innate immune genes in intestinal epithelial cells, and to prevent sensitization to food allergens in mice (44, 45). In the placebo group the observed marked increase in *F. prausnitzii* might have been influenced by the fiber-rich cellulose in the placebo capsules. Cellulose, generally regarded as inert, may have been food for growth for bacterial species thriving on dietary fiber, such as the Firmicute *Butyrivibria crossus* (46, 47).

Our study was adequately powered for and participants had been selected on the primary outcome measures, SBP and DBP, allowing also for exploration by subgroups, and providing sufficient data for analysis of central hemodynamic measures such as central blood pressure, and pulse wave velocity, a marker for arterial stiffness.

However, assessments of the effect of aged garlic extract on most secondary outcome measures, including inflammatory markers, Vitamin B12 levels, and gut microbiota was exploratory, and statistical analysis was limited by smaller numbers in the subgroups of participants with high or low levels of these markers at baseline.

In addition, longer trials may be needed to provide statistical evidence of a potential correlation between blood pressure and gut microbiota, as a previous small human study suggested demonstrating a significant difference in microbial richness and diversity between hypertensives and normotensives 15. In our trial all participants were hypertensives, and a period of 3 months may not be sufficient to achieve a statistically significant microbial change representative of a long-term normotensive environment.

## Conclusions

In summary, our trial consolidated current evidence for Kyolic aged garlic extract to be effective in reducing blood pressure in individuals with uncontrolled hypertension similarly to standard blood pressure medication. Kyolic aged garlic extract is highly tolerable, and can be taken safely in addition to other standard blood pressure medication. Our trial provided new evidence for Kyolic garlic to improve central hemodynamic measures and arterial stiffness, regarded as important predictors for cardiovascular disease.

Our trial is the first to explore the effect of Kyolic aged garlic extract on gut microbiota in hypertensives, with promising results toward an increase in microbial richness and diversity, and a marked increase in the beneficial and immune stimulating bacteria, *Lactobacillus* and *Clostridia* species, within a 3-month period. Together with the potential lowering of inflammation, Kyolic garlic provides beneficial effects on several levels important for cardiovascular health.

Further larger and longer-term studies are warranted to assess the potential of Kyolic aged garlic extract on gut microbiota, inflammation and immunity. Moreover, it would be of interest to explore individuals' responsiveness to aged garlic extract, by investigating underlying dietary and genetic factors such as vitamin B6, folate levels, in addition to vitamin B12.

## Ethics Statement

This study was carried out in accordance with the recommendations of the National Health and Medical Research Council (NHMRC) registered NIIM Human Research Ethics Committee (HREC) with written informed consent from all subjects in accordance with the Declaration of Helsinki. The protocol was approved by the NIIM HREC.

## Author Contributions

KR and AS conceptualized the study. KR acquired funding and oversaw data collection by NT. KR, and NT undertook data analysis. KR prepared the manuscript with contributions from co-authors. All authors approved the final version.

### Conflict of Interest Statement

The authors declare that the research was conducted in the absence of any commercial or financial relationships that could be construed as a potential conflict of interest.
