# Supplementary material for: The Effect of Kyolic Aged Garlic Extract on Gut Microbiota, Inflammation, and Cardiovascular Markers in Hypertensives: The GarGIC Trial
Source: Front Nutr. 2018 Dec 11;5:122. doi: 10.3389/fnut.2018.00122 (PMC6297383; doi:10.3389/fnut.2018.00122)
Supplement: Supplementary file 1 [file Table_1.pdf]

Supplementary Material

The Effect of Kyolic Aged Garlic Extract on Gut Microbiota, Inflammation and Cardiovascular Markers in Hypertensives: the GarGIC trial

Karin Ried*, Nikolaj Travica and Avni Sali

*** Correspondence:** Corresponding Author: karinried@niim.com.au

Supplementary Table S1:

GarGIC trial – Gut microbiota analysis:

Relative abundance in colony forming units per gram of stool (CFU/g) by bacteria groups and phyla, change between baseline and 12 weeks

Phylum	Variable	Group	N	Mean CFU/g	Std. Deviation CFU/g	Std. Error Mean CFU/g	Mean diff g/p p-value
**Bacteroidetes**	v41_Bac1_change Bacterioides-Prevotella group	garlic	21	-102190476	1579602849	344697601	
		placebo	25	94720000	1423822570	284764514	0.6
	v41_Bac2_change Bacterioides vulgatus	garlic	21	660533333	7534138289	1644083762	
		placebo	25	-227520000	5976610007	1195322001	0.6
	v41_Bac3_change Barnesiella spp	garlic	21	-131028571	691445802	150885844	
		placebo	25	14496000	382866408	76573282	0.4
	v41_Bac4_change Odoribacter spp	garlic	21	52238095	191664526	41824628	
		placebo	25	40052000	97578811	19515763	0.8
	v41_Bac5Prev_change Prevotella spp	garlic	21	53119524	238064646	51949965	
		placebo	25	1583600	10588480	2117696	0.3
	v41_sumBac_change Bacteroidetes phylum	garlic	21	532671905	9676812414	2111653589	
		placebo	25	-76776400	6762622454	1352524491	0.8
**Firmicutes**	v41_Firm1_change Anaerotruncus colihominis	garlic	21	8646667	64528238	14081216	
		placebo	25	4743600	18105580	3621116	ns
	v41_Firm2_change Butyrivibrio crossotus	garlic	21	81019	879640	191953	
		placebo	25	1013404	4984884	996977	ns
	v41_Firm3_change Clostridium spp	garlic	21	5342380952	20033283280	4371620812	
		placebo	25	-1163200000	12446397310	2489279462	ns
	v41_Firm4_change Coprococcus eutactus	garlic	21	10557619	58215731	12703714	
		placebo	25	6038400	50456696	10091339	ns
	v41_Firm5_change Faecalibacterium prausnitzii	garlic	21	-428571429	8842975340	1929695422	
		placebo	25	6518640000	16669795880	3333959175	0.08; adjusted for baseline p=0.1
	v41_Firm6_change Lactobactillus spp	garlic	21	1567428571	5950759965	1298562285	
		placebo	25	282720000	3476761706	695352341	ns
	v41_Firm7_change Pseudoflavonifractor spp	garlic	21	-2928571	499533440	109007133	
		placebo	25	-27920000	291081163	58216233	ns
	v41_Firm8_change Reseburia spp	garlic	21	-316666667	5421280599	1183020415	
		placebo	25	-232400000	4886580502	977316100	ns
	v41_Firm9_change Ruminococcus spp	garlic	21	26095238	709076787	154733241	
		placebo	25	-71440000	771168706	154233741	ns
	v41_Firm10_change Veillonella spp	garlic	21	14233333	55290490	12065374	
		placebo	25	7219200	36777464	7355493	ns
	v41_sumFirm_change Firmicutes phylum	garlic	21	6221280543	34063630630	7433293609	
		placebo	25	5325405404	24947338920	4989467784	ns

Phylum	Variable	Group	N	Mean CFU/g	Std. Deviation CFU/g	Std. Error Mean CFU/g	Mean diff g/p p-value
**Actinobacter**	v41_Actino1_change Bifidobacterium spp	garlic	21	-521095238	2604558118	568361177	
		placebo	25	-269880000	2968635465	593727093	ns
	v41_Actino2_change Bifidobacerium longum	garlic	21	45190476	347404464	75809869	
		placebo	25	31840000	226644303	45328861	ns
	v41_Actino3_change Collinsella aerofaciens	garlic	21	323157143	1320592147	288176832	
		placebo	25	97560000	776905189	155381038	ns
	v41_sumActino_change Actinobacter phylum	garlic	21	-152747619	3711925194	810008485	
		placebo	25	-140480000	3478970539	695794108	ns
**Proteobacter**	v41_Proteo1_change Desulfovibrio piger	garlic	21	19046190	62367344	13609670	
		placebo	25	6889600	37611723	7522345	ns
	v41_Proteo2_change Escherichia coli	garlic	21	27599524	297120670	64837046	
		placebo	24	13758333	70068091	14302589	ns
	v41_Proteo3_change Oxalobacter formigenes	garlic	21	8275714	30660680	6690709	
		placebo	25	-424800	15455530	3091106	ns
	v41_sumProteo_change Proteobacteria phylum	garlic	21	54921429	342656644	74773810	
		placebo	25	5312800	114886366	22977273	ns
**Euryarchaeota**	v41_methano_change Methanobrevibacter smithii	garlic	21	20109524	115121926	25121664	
		placebo	25	-16509200	59364747	11872949	ns
**Fusobacteria**	v41_Fuso_change Fusobacterium spp	garlic	21	57624	202365	44160	
		placebo	25	-72876	206612	41322	0.04, adjusted for baseline p=0.08
**Verrucomicrobia**	v41_Akkerman_change Akkermansia muciniphila	garlic	21	-5758571	31897695		
	v41_Akkerman_change	placebo	25	-4228000	26078373		ns

V1= visit 1= baseline = 0 weeks
V4= visit 4 = 12 weeks
V41 = change between v4 and v1
ns = not significant
CFU/g = Colony Forming Units per gram stool
